# Development and Characterization of Sulfasalazine Cubosomes for Potential Transdermal Drug Delivery

**DOI:** 10.2174/0122117385269522231113041029

**Published:** 2024-02-15

**Authors:** Mekha Mathew, Anasuya Patil, Hemanth G

**Affiliations:** 1 Department of Pharmaceutics, KLE College of Pharmacy, Rajajinagar, Bengaluru, India

**Keywords:** Cubosomes, sulfasalazine, rheumatoid arthritis, glyceryl monooleate, poloxamer 407, transdermal delivery

## Abstract

**Background:**

Rheumatoid arthritis is indeed a constant, progressive autoimmune disease that acts on the synovial membrane, distinguished by joint pain, swelling, and tenderness. Sulfasalazine belongs to BCS Class IV having low solubility and low permeability. To overcome the issue and provide a localized effect Cubosomes were chosen for the transdermal drug delivery system.

**Objectives:**

The primary objective of this investigation was to pass on sulfasalazine-loaded cubosomes over the skin to treat rheumatoid arthritis. On the way to overcome this issue of oral sulfasalazine and provide localized effect, Cubosomes were chosen for the transdermal drug delivery system.

**Methods:**

Sulfasalazine-loaded cubosomes were prepared by the top-down method using GMO and Poloxamer 407. Different concentrations of lipid and surfactant were used in the formulation using 3^2^ full factorial designs. The prepared formulations were assessed for p.s, z,p, %EE, FTIR, SEM, *in-vitro* release, *ex-vivo* permeation, and deposition studies with pH 7.4 phosphate buffer saline.

**Results:**

The particle size varies between 65 nm to 129 nm, while the negative zeta potential ranged from -18.8 mV to -24.8 mV. The entrapment efficiency was between 87% and 95%. The formulations' *in-vitro* drug release was carried out for 12 hours. The optimized formulation showed a controlled release of sulfasalazine and better *ex-vivo* permeation and deposition properties than sulfasalazine suspension.

**Conclusion:**

Overall study findings support the possibility of applying transdermal sulfasalazine-loaded cubosomes to alleviate rheumatoid arthritis.

## 1 INTRODUCTION

The synovial joint lining is primarily impacted by the chronic inflammatory autoimmune condition identified as rheumatoid arthritis (RA), which is manifested by joint pain, inflammation, stiffness, and synovial membrane destruction. The prevalence of this illness in the population ranges from 0.5% to 1%, and it affects women more frequently than men and the elderly in particular [1].

The immune system of the body targets its own normal cells, which leads to the emergence of rheumatoid arthritis. There is yet no precise cause for RA. Rheumatoid arthritis is thought to develop when hereditary and environmental variables combine to cause immunological alterations that result in inflammatory arthritis [2]. The secretion of pro-inflammatory cytokines such as interleukin IL-1, IL-6, and TNF- α leads to cartilage degeneration, bone erosion, and joint abnormalities [3].

Disease-modifying anti-rheumatic medicines (DMARDs) are a type of medication primarily used to treat a number of inflammatory arthritis, including rheumatoid arthritis, psoriatic arthritis, osteoarthritis, spondyloarthritis, and arthritis-related inflammatory bowel disease [4]. Sulfasalazine is a U.S. FDA-approved disease-modifying anti-rheumatic medicine (DMARD) which is commonly used as its initial course of treatment for rheumatoid arthritis. It belongs to BCS Class IV medication with minimal permeability and solubility. Oral therapy of sulfasalazine causes gastric irritation, indigestion, and heartburn. To avoid such complications of sulfasalazine oral treatment, the transdermal route of administration is preferred. Owing to its anti-inflammatory action of sulfasalazine, it reduces joint pain, and inflammation and controls the progression of the disease with minimum side effects [5].

Cubosomes are crystalline liquid particles with certain amphiphilic lipids and surfactants arranged in particular concentrations in an aqueous medium. Cubosomes are composed of two internal water channels and curved bi-continuous lipid bilayers arranged in a honeycomb pattern in three dimensions with a size range of 50-600 nm [6]. Because of their special qualities, such as thermodynamic endurance, bio-adhesion, capability to encapsulate hydrophilic, hydrophobic, and amphiphilic compounds, and the potential for controlled release, cubosomes have been considered attractive carriers for a broad range of routes of administration [7]. The amphiphilic nature of cubosomes interacts with the lipids present in the skin’s outermost layer, called the stratum corneum. This interaction helps the cubosomes to penetrate effectively through the lipid layer and reach essential skin layers. Cubosomes can release drugs in a controlled manner or hasten the penetration of drugs through the stratum corneum. Thus it provides controlled delivery of drugs over an extended period, by avoiding recurrent application and improving patient compliance [8]. Cubosomes can be prepared using surfactants like Poloxamer 407 and biodegradable lipids like glyceryl mono-oleate (GMO) [9]. Glyceryl monooleate (GMO) is an ecological substance that can form cubic particles with high viscosity when placed in an aqueous medium. This cubic structure is beneficial for drug delivery systems because it can encapsulate drugs and provide a controlled release pattern. On the other hand, Poloxamer 407 is a synthetic copolymer that has some molecular relationship to glyceryl monooleate (GMO). Poloxamer 407 was added to stabilize the cubic shape of the cubosomes and prevent particle clumping.

The formulation and analysis of sulfasalazine-loaded cubosomes were the principal objectives of this work. *Ex-vivo* investigation has been conducted in order to compare the selected cubosomal formulation with sulfasalazine suspension. In this work, sulfasalazine-loaded cubic particles were created in order to improve transdermal drug distribution and therapeutic efficacy while decreasing side effects. As a result, the preparation of sulfasalazine-loaded cubosomes and an analysis of the parameters were the goals of this study.

## 2 MATERIALS AND METHODS

Sulfasalazine, Glyceryl monooleate (GMO), and Poloxamer 407(P407) were acquired from Yarrow Chemicals, Mumbai. The dialysis membrane was procured from Hi-Media Pharmaceuticals Pvt. Ltd. The analytical grade chemicals and reagents used in the study were all procured from commercial providers.

### 2.1 Preparation of Sulfasalazine-loaded Cubosomes

Sulfasalazine-loaded cubosomal formulations were prepared by using the Top-Down technique. Nine different batches were prepared by varying the concentration of Glyceryl monooleate (GMO) and Poloxamer 407. The drug was completely dissolved in ethanolic solution and added to GMO and poloxamer 407 mixture maintained at 70°C. The above mixture is added slowly to the aqueous phase maintained at 70°C and then mixed using a magnetic stirrer at 1500 rpm keeping temperature constant [10]. The solution was homogenized at 10000 rpm for 20 minutes (IKA ^®^ T18 ultra turrax) homogenizer followed by sonication by means of (Labman™) probe sonicator for 20 minutes to form a dispersion of sulfasalazine. The prepared formulations were further characterized.

### 2.2 Optimization of Cubosomal Formulation

Sulfasalazine-loaded cubosomes were statistically optimized by using three levels and two factors (3^2^) of full factorial design [11]. The optimization was performed by Minitab^®^21 software by varying the concentration of independent variables lipids as X_1_ (2.5% - 4.5%w/v), and surfactant as X_2_ (0.5% - 1.5% w/v). The effect of GMO and P407 influences the p.s (Y1), z.p, and %EE (Y2). This design depicted 9 experimental runs. Lower particle size and greater entrapment efficiency were employed to determine the optimized formulation composition. Later the statistical analysis was evaluated to examine the impact of several factors (X_1_, X_2_) on (Y_1_, Y_2_) [12]. The formulation table of cubosomes is shown in Table **1**.

## 3 EXPERIMENTAL

### 3.1 Particle Size, Poly-dispersity Index (PDI) & Zeta Potential

At 25°C, the prepared cubosomal preparations were assessed for p.s, PDI, and z.p by Malvern Zetasizer. One milliliter of the sample from all selected formulations was obtained prior to the measurement and diluted in ten milliliters of distilled water. The samples were kept in a glass cuvette, and measurements were taken at a 90° fixed angle [9].

### 3.2 Entrapment Efficiency

The ultra-centrifugation method was employed to measure % of drug entrapped [13]. Centrifugation was performed on the cubosomal formulations having 1 mg equivalent weight of sulfasalazine at 4°C for 45 minutes at 8000 rpm. In order to analyze the absorbance under an ultraviolet-visible spectrophotometer at 359 nm, a supernatant solution containing free drug was pipetted and diluted to 10ml with a phosphate buffer solution pH 7.4 [14]. The following equation was used to determine the %entrapment efficiency.


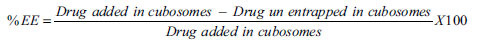


### 3.3 FT-IR Spectroscopy

The interaction between drugs and excipients was determined by the FT-IR study. The main peaks of the drug, excipients, physical mixture, and formulation were performed to check the compatibility of the formulation composition. The samples were generated in potassium bromide discs, and the spectrum was obtained using a 400 - 4000 cm^-1^ resolution JASCO FT-IR 460 plus spectrophotometer [15].

### 3.4 Form and Superficial Composition

The form and superficial anatomy of sulfasalazine-loaded cubosomes have been investigated by scanning electron microscopy (SEM). The cubosomes were diluted 100x and over a cover slip, a tiny amount of the material was evenly applied and left to air dry. Then the sample was covered with a VEGA3 TESCAN sputter and photomicrographs were captured at various magnifications [16].

### 3.5 *In-vitro* Release

The dialysis approach was used to conduct *in-vitro* release. The release study utilizes a membrane for dialysate. Before the release trials, the dialysis membrane was immersed in distilled water. 1mg equivalent weight of the sulfasalazine-loaded cubosomal formulations was poured into a membrane utilized for dialysis membrane that is knotted at endings. The membrane was immersed in a container with 100 milliliters of phosphate buffer solution pH 7.4 with 1% tween 80 while being stirred magnetically at 50 rpm and 35±1°C. At predetermined intervals of 0.5, 1, 2, 3, 4, 6, 8, and 12 hours, the samples were taken out and refilled with the same buffer. Sulfasalazine-loaded cubosomal formulation is compared with a pure drug suspension equivalent to 1 mg sulfasalazine respectively [17, 18]. The concentration of sulfasalazine released from the sulfasalazine-loaded cubosomes was calculated by a UV spectrophotometer (Shimadzu UV-1800) at 359 nm. The release of sulfasalazine through the cubic phase was identified by plugging the release rate data into the corresponding equations for zero order, first order, the Higuchi model, the Korsemeyer-Peppas model, and the Hixson-Crowell model.

### 3.6 *Ex-vivo* Permeation Studies

The FDC was used to conduct *ex-vivo* skin permeation tests by snake-shed skin. The cubosomal formulation was compared with sulfasalazine suspension. The Franz diffusion cell was filled with the snake skin, and the receptor chamber contained 10 mL of phosphate buffer solution pH 7.4 with 1% tween 80. The snake shed skin was covered with 1ml of the cubosomal formulation, and the receptor media was kept at 35±1°C while being agitated at 30 rpm. To keep the sink state, 1 ml of the sample was taken and refilled with new buffer at intervals of 0.5, 1, 2, 3, 4, 6, 8, and 12 hours [19, 20]. Permeation of sulfasalazine across the skin was quantified by a UV spectrophotometer at 359nm.

### 3.7 Study of Skin Deposition

The above skin is carefully removed and cleaned with fresh buffer. Using 5 ml of ethanol and sonicate for 12 minutes, the sulfasalazine that had been retained in the skin was removed. A filter was used to filter the samples [21, 22]. The percentage of sulfasalazine accumulated in the snake-shed skin was determined by a UV spectrophotometer at 359nm.

## 4 RESULTS AND DISCUSSION

### 4.1 Formulation Design

A 3^2^-full factorial design was used to optimize the cubosomal formulation. The concentrations of poloxamer 407 and glyceryl monooleate remained X1 and X2. Y1, Y2, and Y3 have been identified as p.s, z.p, and %EE [23]. Based on the outcomes of the inputs, the formulations were optimized.

### 4.2 Particle Size

Malvern apparatus was used for assessing the p.s, PDI, and z.p of sulfasalazine-loaded cubosomes. All of the formulations' particle sizes were discovered to fall between 65nm and 129 nm, and the particle size distribution graph is depicted in Table **1** and Fig. (**1**). All of the formulations' PDI values were discovered to be below 1. The PDI score shows that the cubosomal formulations’ particle size is uniformly distributed. The formulations’ particle size depended on the concentration of GMO and poloxamer 407. The particle size of cubosomes decreased when the poloxamer 407 level was increased, and it considerably increased when GMO concentration got higher.

P.s was plotted against GMO and Poloxamer 407 in Fig. (**2**).

Regression equation of particle size = 99.67-9.33GMO_2.5-17.00GMO_3.5+26.33GMO_4.5+14.67POLOXAMER-407_0.5-11.67POLOXAMER-407_1.0-3.00POLOXAMER-407_1.5.

### 4.3 Entrapment Efficiency

Sulfasalazine-loaded cubosomes have been shown an entrapment efficiency of between 87% and 95% Entrapment efficiency is directly related to the level of GMO and poloxamer 407 due to improved lipophilic drug solubilization. The SC6 formulation having 3.5% lipid concentration and 1.5% surfactant concentration was shown to have a higher entrapment efficiency than other cubosomal formulations. % EE was plotted against GMO and Poloxamer 407 in Table **1** and Fig (**3**).

The regression equation for entrapment efficiency: 90.600-2.597 GMO_3.5-0.217 GMO_4.5-0.297 POLOXAMER 407_0.5-0.330 POLOXAMER 407_1.0+0.627 POLOXAMER 407_1.5.

### 4.4 Zeta Potential

The zeta potential distribution graph for all 9 cubosomal formulations was achieved and the selected formulation is shown in Fig. (**4**), and it was in a region of -18.8 mV to -24.8 mV. These values depict that the cubosomes possess a neutral zeta potential due to poloxamer 407 and GMO usage. The high zeta potential value prevents aggregation of particles due to the electric repulsion. Z.p was plotted against GMO and Poloxamer 407 in Fig. (**4**).

### 4.5 Fourier Transform Infrared Spectroscopy

FTIR studies show the peak of sulfasalazine at wavelength 3074.94 cm^-1^, 1618.95 cm^-1^, and 1394.28 cm^-1^ for the functional group’s OH, NH, O=S=O. FTIR spectra of GMO represent the peak at 3449 and 1740 cm^-1^ for OH, C=O functional group, although poloxamer 407 at 1742 cm^-1^, 1468 cm^-1^, and 1339 cm^-1^ for C=O, CH3, C=C functional group respectively. The majority of the functional group peaks present show no evidence of drug-polymer interaction [24]. Fig. (**5**) represents the infrared spectra of sulfasalazine, glyceryl monooleate, poloxamer 407, physical mixture, and optimized formulation (SC6).

### 4.6 Cubosomal Morphology

SEM analysis was used to assess the cubosomes' form and superficial anatomy. The optimized formulation's findings (SC6) demonstrated that the particles have an irregular cubic shape with smooth surfaces and confirmed that they fall within the nano range. Fig. (**6**) indicates the SEM image of the optimized formulation (SC6).

### 4.7 *In-vitro* Release

Sulfasalazine was released from developed cubosomes over the course of 12 hours utilizing a dialysis membrane. Drug diffusion across the cubosomes is the framework for the drug release process from sulfasalazine-loaded cubosomes. After calculating the drug release pattern of all 9 formulations for 12 hours, SC6 had better drug release than other formulations. Thus SC6 is considered as the finalized formulation having 85.92% release as it showed sustained release for 12 hours owing to entrapment of the drug. The release profile of all 9 formulations is depicted in Fig. (**7**). Sulfasalazine released through the cubic phase was identified by plugging the release rate data into the corresponding equations for zero order, first order, the Higuchi model, the Korsemeyer-Peppas model, and the Hixson-Crowell model [25]. The Higuchi model achieved the best fit with the selected formulation which is depicted in Table **2**.

### 4.8 *Ex-vivo* Permeation Studies

Snake shed skin is used for permeation study as it has structural similarity with human skin. The amount of sulfasalazine diffused through the skin is compared with the selected formulation (SC6) and sulfasalazine suspension. The finalized formulation showed 62.16% drug release from sulfasalazine-loaded Cubosomes for up to 12 hours than sulfasalazine suspension. The lipid (GMO) used in the preparation act as permeation enhancers. The amount of sulfasalazine permeated from the finalized formulation (SC6) and drug suspension is depicted in Fig. (**8**). Owing to the structural similarity between cubosomes and epithelial cells, cubosomes can easily penetrate through the skin, resulting in enhanced drug bioavailability. Ethanol which is used as a solvent increases the solubility and permeability of the drug.

### 4.9 Study of Skin Deposition

The selected formulae SC6 showed the maximum skin deposition. Than sulfasalazine suspension. C6 formulation is capable of 3 times more deposition compared to the drug suspension. The percentage of sulfasalazine deposited from SC6 and drug suspension is depicted in Fig. (**9**). The GMO used provides drug retention and permeation effect and facilitates transdermal drug delivery of the drug by a noninvasive route.

## CONCLUSION

The above outcomes confirmed that sulfasalazine-loaded cubosomes enhance the efficacy of the drug sulfasalazine in terms of solubility and permeability. Sulfasalazine-loaded cubosomes have an irregular cubic-shaped particle and were found to be within the Nano range. By virtue of their unique structure, cubosomes have better entrapment efficiency (≥87%) and showed sustained release of the drug. Sulfasalazine-loaded cubosomes showed better *ex-vivo* skin permeation and deposition properties compared to sulfasalazine suspension. Therefore, from this study, we can conclude that sulfasalazine-loaded cubosomes provide promising transdermal delivery for the treatment of rheumatoid arthritis.

## Figures and Tables

**Fig. (1):**
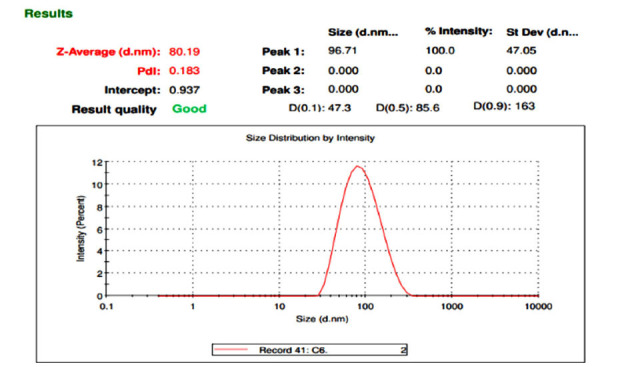
Particle size graph of SC6.

**Fig. (2):**
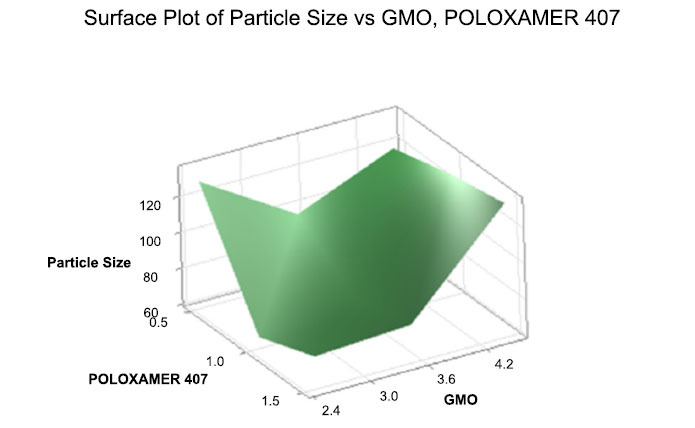
3D Surface plot of particle size against GMO and P- 407.

**Fig. (3):**
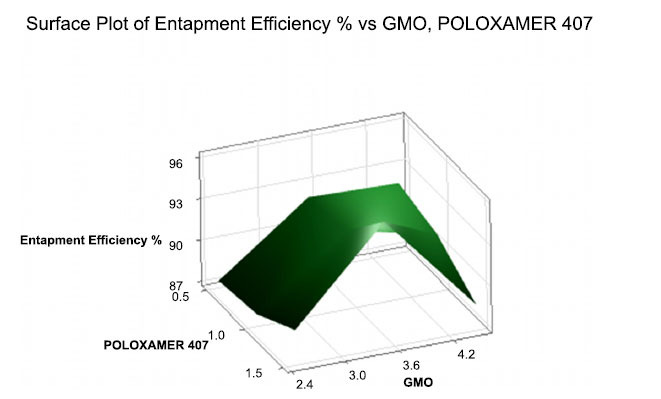
3D Surface plot of particle size against GMO and P- 407.

**Fig. (4):**
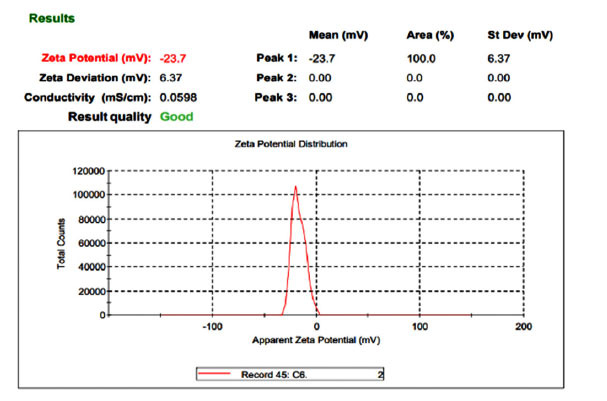
Zeta potential graph of SC6.

**Fig. (5):**
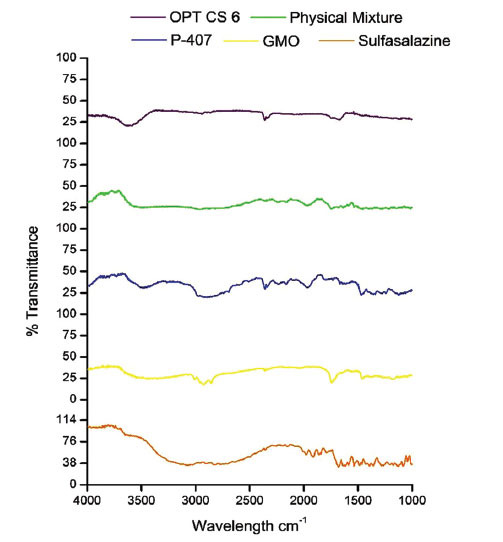
FT-IR graph of sulfasalazine, glyceryl monooleate, poloxamer 407, physical mixture, and optimized formulation (SC6).

**Fig. (6):**
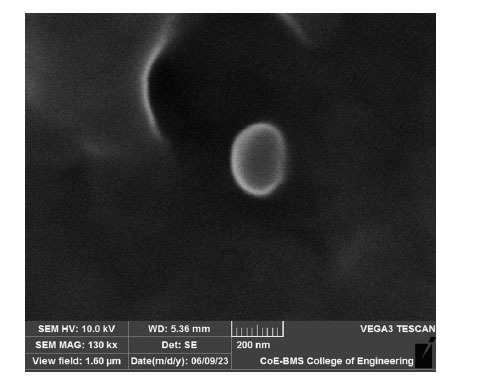
SEM image of the optimized formulation (SC6).

**Fig. (7):**
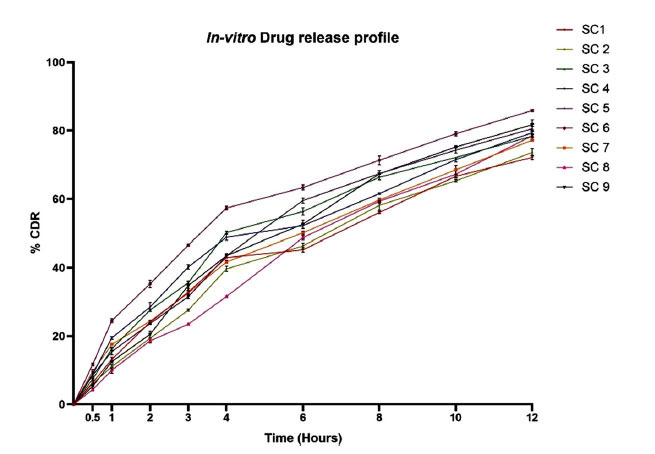
% Cumulative drug release profiles of SC1 to SC9.

**Fig. (8):**
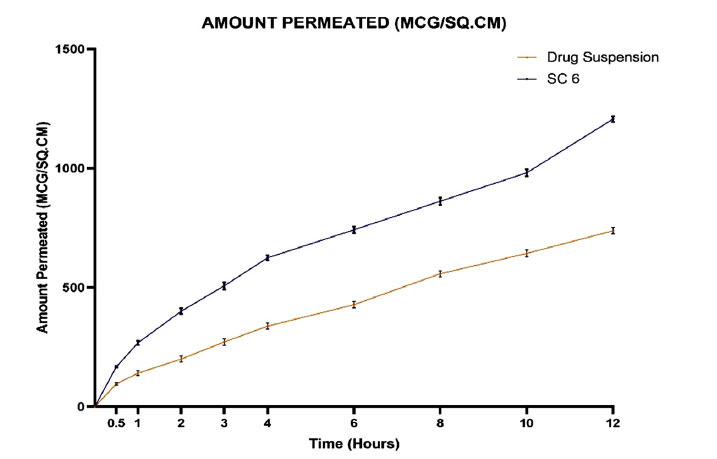
Percentage of drug permeation from plain drug and cubosome (SC6).

**Fig. (9):**
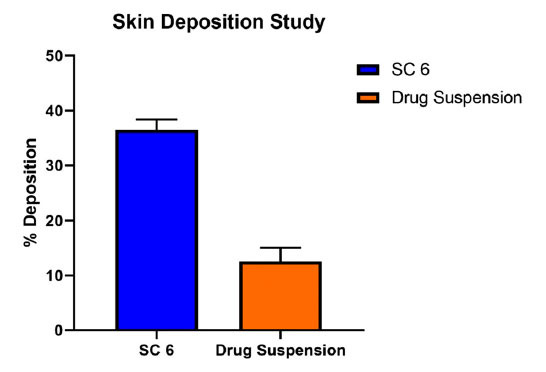
The percentage of sulfasalazine deposited on snake shed skin of SC6 and drug suspension.

**Table 1:** Formulation table of sulfasalazine-loaded cubosomes.

**Formulation Code**	**Drug (mg)**	**Independent Variables**		**Dependent Variables**	
		**X_1_ (GMO)**	**X_2_ (P407)**	**Y_1_ (P.S) nm**	**Y_2_ (% EE)**
SC1	50	2.5%	0.5%	128±0.5	83.18±0.69%
SC2	50	2.5%	1%	64±0.5	84.86±0.73%
SC3	50	2.5%	1.5%	78±0.5	86.47±1.04%
SC4	50	3.5%	0.5%	95±0.5	84.27±0.84%
SC5	50	3.5%	1%	72±1.1	87.91±0.33%
SC6	50	3.5%	1.5%	80±1.7	91.02±0.57%
SC7	50	4.5%	0.5%	119±0.5	86.97±0.96%
SC8	50	4.5%	1%	127±1.1	88.72±0.58%
SC9	50	4.5%	1.5%	133±0.5	90.83±0.46%

**Table 2:** Different release kinetics of SC6.

**Formulation**	**Models**	**R^2^**
SC 6	Zero order	0.7264
	First order	0.9719
	Higuchi	0.9873
	Korsemeyer-peppas	0.9857
	Hixon-crowell	0.9323

## Data Availability

The data and supportive information are available within the article.

## LIST OF ABBREVIATIONS

% EE: Entrapment Efficiency
FT-IR: Fourier Transform Infrared Spectroscopy
p.s: Particle Size
PDI: Poly-Dispersity Index
SEM: Scanning Electron Microscopy
z.p: Zeta Potential
